# *TFE3*-Rearranged and *TFEB*-Altered Renal Cell Carcinomas: Molecular Landscape and Therapeutic Advances

**DOI:** 10.3390/cancers18060958

**Published:** 2026-03-16

**Authors:** Mikel Portu, Mario Balsa, Maria Cotaina, Georgia Anguera, Xavier García del Muro, Ferran Algaba, Pablo Maroto

**Affiliations:** 1 Medical Oncology Department, Hospital de la Santa Creu i Sant Pau, 08041 Barcelona, Spain; 2 Medical Oncology Department, Catalan Institute of Oncology (ICO), 08908 L’Hospitalet de Llobregat, Spain; 3 Pathology Department, Fundació Puigvert, 08025 Barcelona, Spain

**Keywords:** MiT-RCC, translocation renal cell carcinoma, *TFE3*, *TFEB*, gene fusion, immune checkpoint inhibitor, tyrosine kinase inhibitor, precision oncology

## Abstract

Renal cell carcinomas (RCCs) driven by *TFE3* rearrangement or *TFEB* alteration (MiT-RCC) account for up to 40% of pediatric RCCs but are rare in adults. MiT-RCC includes fusion-driven tumors with *TFE3* or *TFEB* rearrangements (translocation RCC, tRCC) and *TFEB*-amplified RCC. Morphologic heterogeneity and historical exclusion from trials have limited evidence-based management. We reviewed the literature through January 2026 to summarize molecular biology, pathology, clinical behavior, and systemic therapy. MiT-RCC comprises biologically distinct entities: *TFEB*-rearranged tumors are often indolent in younger patients, whereas *TFEB*-amplified RCC, frequently co-amplifying *VEGFA*, behaves aggressively in older adults. In *TFE3*-rearranged RCC, fusion partner influences prognosis. Paradoxically, *ASPSCR1*::*TFE3* fusions have the poorest natural history, yet fusion-annotated cohorts suggest these tumors may derive particular benefit from immune checkpoint inhibitor (ICI) plus VEGF receptor tyrosine kinase inhibitor (VEGFR-TKI) combinations. Diagnostic advances including GPNMB immunohistochemistry, *TRIM63* RNA in situ hybridization, and sequencing-based fusion panels improve detection of cryptic alterations. First-line ICI + VEGFR-TKI combinations are increasingly favored for metastatic tRCC in eligible patients, while optimal management of *TFEB*-amplified RCC remains uncertain.

## Simple Summary

MiT family renal cell carcinomas (MiT-RCCs) are rare kidney cancers defined by alterations in the *TFE3* or *TFEB* genes. Tumors driven by gene fusions tend to affect children and young adults, whereas tumors with *TFEB* amplification more often occur in older adults and can be aggressive. These cancers are not a single entity but a group of molecular subtypes with different clinical behavior, from indolent to rapidly progressive. Recent studies suggest that combining immunotherapy with anti-angiogenic targeted therapy can produce meaningful responses in metastatic fusion-driven disease, although evidence remains limited because these patients have often been excluded from clinical trials. Accurate diagnosis increasingly requires molecular testing in addition to standard pathology. This review summarizes tumor biology, diagnostic approaches, and treatment evidence to support clinical decision-making in this evolving disease spectrum.

## 1. Introduction

Molecularly defined renal cell carcinomas (RCCs) represent a major category in the 2022 World Health Organization (WHO) classification and include the subgroup historically termed “MiT family translocation RCC” or “translocation RCC” (tRCC). These tumors are defined by chromosomal aberrations involving transcription factors of the microphthalmia-associated transcription factor (MiT) family, most commonly Transcription Factor E3 (*TFE3*) at Xp11.2 (hence the legacy term “Xp11.2 translocation RCC” for *TFE3*-rearranged tumors) or Transcription Factor EB (*TFEB*) at 6p21 [1,2]. Based on this genomic characterization, the 2022 WHO classification identifies two specific molecular entities: RCC with *TFE3* rearrangement and RCC with *TFEB* alteration [1]. Importantly, the *TFEB* alteration category encompasses both *TFEB*-rearranged tumors (classically t(6;11)/*MALAT1*::*TFEB*) and *TFEB*-amplified RCC, which are now recognized as biologically and clinically distinct drivers [1,3,4].

In this review, we use the term MiT-RCC as an umbrella designation for renal cell carcinomas with *TFE3* rearrangement or *TFEB* alteration (including both *TFEB*-rearranged and *TFEB*-amplified tumors). For clarity, we use translocation RCC (tRCC) only when referring to fusion-driven MiT-RCC (*TFE3*-rearranged and *TFEB*-rearranged tumors) and do not apply “tRCC” to *TFEB*-amplified RCC. Because *TFEB* amplification was recognized and separated conceptually later than the classic “MiT family translocation RCC” literature, many older series labeled “MiT-RCC” or “tRCC” are composed predominantly of fusion-driven tumors. Throughout this review, we therefore label those datasets as tRCC unless *TFEB* amplification was explicitly included or analyzed.

From a therapeutic standpoint, the rarity of MiT-RCC and its underrepresentation (and frequent exclusion) from prospective RCC trials have historically limited evidence-based treatment recommendations, leading to extrapolation from other non-clear-cell RCC subtypes [5].

The epidemiology varies significantly by age and molecular subtype. In pediatric and young adult RCC cohorts, MiT translocation RCC can account for ~40% of cases and is composed predominantly of *TFE3*-rearranged tumors (with a smaller minority of *TFEB*-rearranged cases) [6]. In contrast, *TFE3*-rearranged RCC is rare among unselected adult RCC, reported at ~1% in a large consecutive adult surgical series [7]. Across adult series, there is a slight female predominance (female-to-male ratio ~1.5–2:1) [5,8]. Recent genomic analyses provide a mechanistic explanation for this sex bias: *TFE3* fusions can arise from either the active X chromosome (Xa) or the inactive X chromosome (Xi), and Xi-derived events are associated with partial reversal of X-inactivation/chrXp reactivation that may enable expression of the oncogenic fusion transcript. Because Xi:autosome translocations are intrinsically female-specific, recurrent access to Xi for *TFE3* fusion formation provides a genetic basis for the observed sex bias [8].

Within the *TFEB* alteration category, clinical behavior depends on the underlying mechanism. *TFEB*-rearranged tumors (t(6;11)) tend to occur in younger patients and often follow an indolent course [3,5,9], whereas *TFEB*-amplified RCC is molecularly and clinically distinct, typically occurring in older adults (median age ~65 years) and frequently displaying aggressive behavior [4]. To date, the best-documented epidemiologic association for the development of *TFE3*-rearranged RCC is prior exposure to cytotoxic chemotherapy, particularly during childhood [5,10]. Beyond chemotherapy-associated cases, consistent epidemiologic risk factors have not been established.

Histopathologic diagnosis can be challenging because these tumors are heterogeneous and can resemble other RCC subtypes. They may show nested, alveolar, or papillary architectures, eosinophilic cytoplasm, and frequent psammoma bodies [5]. While immunohistochemistry (IHC) can detect nuclear overexpression of *TFE3* or *TFEB* proteins, sensitivity and specificity are imperfect and results can be affected by pre-analytic and analytic variables [2,5]. Therefore, confirmatory molecular testing, most commonly fluorescence in situ hybridization (FISH), with RT-PCR and/or next-generation sequencing (NGS) used depending on local assay availability remains the reference standard to demonstrate the defining gene rearrangements or amplifications [11,12].

Clinically, MiT-RCC may present similarly to conventional RCC (hematuria, flank pain, incidental findings), but adult cases frequently present at advanced stages and can behave aggressively. In retrospective series, cancer-specific survival has been reported to be broadly similar to clear-cell RCC (ccRCC) and poorer than papillary RCC, although outcomes are heterogeneous and influenced by stage and molecular subtype [5,7]. Compared with ccRCC, *TFE3*-rearranged tumors have shown higher recurrence rates (50% vs. ~19% in one series) and shorter progression-free survival; multivariate analyses have identified *TFE3* rearrangement as an independent adverse prognostic factor for recurrence after adjusting for tumor size and stage [13].

Emerging data suggest that fusion partner identity in *TFE3*-rearranged RCC is an important determinant of clinicopathologic phenotype and prognosis and may also influence outcomes on modern immune checkpoint inhibitor (ICI)–tyrosine kinase inhibitor (TKI) combinations, reinforcing the need for precise molecular classification [14,15,16]. This review summarizes current knowledge of tumor biology, diagnostic approaches, clinical behavior, and systemic treatment of *TFE3*-rearranged and *TFEB*-altered RCC to inform clinical decision-making and highlight priorities for future research in this rare disease spectrum.

Terminology note: When discussing specific studies, we adopt the original investigators’ terminology and specify, where reported, whether cohorts included *TFE3*-rearranged, *TFEB*-altered, or mixed populations, and we indicate fusion-partner data (e.g., *ASPSCR1*::*TFE3*) when available. Because *TFE3* rearrangements predominate in the literature, most mechanistic and therapeutic data derive from this subtype, and robust *TFEB*-amplified-specific evidence remains limited to case series.

## 2. Methods

This narrative review synthesizes current evidence on the molecular biology, diagnosis, and treatment of *TFE3*-rearranged and *TFEB*-altered renal cell carcinoma (MiT-RCC). We searched PubMed/MEDLINE, Embase, and the Cochrane Library from database inception through January 2026 using the following search terms: “translocation renal cell carcinoma,” “MiT family RCC,” “*TFE3* rearrangement,” “*TFE3* fusion,” “*TFEB* amplification,” “*TFEB* rearrangement,” “Xp11.2 translocation,” and “t(6;11) renal cell carcinoma.” Boolean operators combined molecular terms (*TFE3*, *TFEB*, MiT, *MITF*) with clinical terms (renal cell carcinoma, kidney cancer, treatment, prognosis, diagnosis). Reference lists of included articles were manually screened to identify additional relevant publications. We also searched ClinicalTrials.gov to identify ongoing or recently completed trials using related terms (translocation RCC, MiT, *TFE3*, *TFEB*) and cross-referenced cited NCT identifiers.

We included peer-reviewed original research articles, case series, and prior reviews that reported on molecular characterization, pathologic diagnosis, clinical outcomes, or systemic treatment of MiT-RCC. Conference abstracts were included when reporting data from prospective clinical trials not yet published in full manuscript form, provided efficacy or survival outcomes were reported with sufficient detail for interpretation. Abstract-only results were considered preliminary and interpreted cautiously. Key recommendations were not based solely on abstract-only data. We excluded single case reports except when describing novel fusion partners or unique therapeutic responses, as well as studies that did not distinguish MiT-RCC from other non-clear-cell RCC subtypes in their analyses. We included English-language publications. We did not identify additional relevant non-English studies during screening.

Given the rarity of MiT-RCC, we did not restrict inclusion by study design. Retrospective cohorts, prospective trials, and translational studies were all considered. For therapeutic evidence, we prioritized prospective clinical trial data where available, followed by large multicenter retrospective series, and then single-institution experiences. When studies reported overlapping patient cohorts, we preferentially cited the most recent or most comprehensive analysis.

Data extraction focused on molecular mechanisms, diagnostic approaches (including method of molecular confirmation, copy-number assessment for *TFEB* amplification, and fusion partner annotation where reported), treatment regimens, response rates, survival outcomes, and prognostic factors. When cohorts were reported as “MiT-RCC” or “translocation RCC” without complete molecular subclassification, we retained the investigators’ terminology and extracted subtype composition and confirmation methods when available. Titles and abstracts were reviewed for relevance, with disagreements resolved through discussion among the authors. No meta-analysis was performed. This review was not registered prospectively, and no formal quality assessment of included studies was conducted, consistent with the narrative review methodology.

## 3. Molecular Landscape of MiT-RCC

### 3.1. MiT/TFE Transcription Factors and Fusion Partners

The MiT/TFE family comprises four basic helix-loop-helix leucine zipper transcription factors: *MITF*, *TFE3*, *TFEB*, and *TFEC*. These proteins are key regulators of lysosomal biogenesis and autophagy and are also involved in cellular metabolism and melanocytic differentiation [17,18,19,20,21,22]. Under physiological conditions, their activity is tightly controlled by nutrient, growth factor, stress, and cell-cycle-sensing pathways [23].

In nutrient-replete cells, mechanistic target of rapamycin complex 1 (mTORC1) is recruited to the lysosomal surface, where it phosphorylates *TFEB* and *TFE3* at conserved serine residues [24]. This phosphorylation promotes 14-3-3 protein binding and cytoplasmic retention, preventing nuclear entry and limiting transcriptional activation of lysosomal and autophagy genes [25,26,27]. During starvation or other stresses that suppress mTORC1 activity, these sites are dephosphorylated, 14-3-3 binding is lost, and *TFEB*/*TFE3* translocate to the nucleus. Additional kinases, including ERK, GSK3, AKT, and CDK4/6, further modulate MiT/TFE localization and stability, linking their activity to mitogenic and cell-cycle cues [25,26,28].

In MiT-RCC, this regulatory circuitry is disrupted by structural genomic alterations that drive constitutive MiT/TFE activation. The most frequent events are chromosomal rearrangements involving *TFE3* at Xp11.2, historically termed “Xp11.2 translocation RCC” in older series and now classified as RCC with *TFE3* rearrangement in the 2022 WHO framework [1,2]. In females, *TFE3* fusions can originate from either the active (Xa) or inactive (Xi) X chromosome, with Xi-origin fusions providing a potential genetic explanation for the observed female predominance [8].

More than 20 *TFE3* fusion partners have been identified, underscoring substantial molecular heterogeneity [2]. These include nuclear RNA-binding and splicing factors (*SFPQ*, *NONO*, *RBM10*, *LUC7L3*, *KHSRP*), splicing-associated proteins (*PRCC*), transcriptional and epigenetic regulators (*MED15*, *KAT6A*), vesicle trafficking proteins (*CLTC*, *ASPSCR1*), noncoding RNA partners (*NEAT1*), and others (*DVL2*, *PARP14*, *GRIPAP1*) [11].

*TFE3* fusion genes are generated by chromosomal rearrangements that join a 5′ partner gene to the 3′ portion of *TFE3*. The resulting fusion transcripts encode chimeric proteins that retain the C-terminal basic helix-loop-helix leucine zipper (bHLH-LZ) DNA-binding and dimerization domains, as well as transactivation capacity of *TFE3*, which are essential for transcriptional activity [29]. In many *TFE3* fusions, the N-terminal regulatory region, which contains multiple phosphorylation sites through which mTORC1 and other kinases contribute to cytoplasmic retention and protein turnover, is replaced by partner-derived sequences [30]. These partner sequences often place *TFE3* under the control of heterologous promoters with high transcriptional activity and, in many cases, oligomerization domains that further stabilize the fusion protein [29]. The net effect is a transcription factor that escapes key regulatory checkpoints that normally restrict MiT/TFE activity and accumulates in the nucleus in a constitutively active state [30].

The second major category, *TFEB*-altered RCC, is defined by genetic alterations of *TFEB* at 6p21. The prototypical lesion is the t(6;11)(p21;q12) *MALAT1*::*TFEB* fusion, first described in children and young adults, which drives high-level *TFEB* expression through promoter swapping [3,9,31]. In addition, a subset of tumors harbors high-level *TFEB* amplification within a 6p21 amplicon that frequently includes *VEGFA* and sometimes *CCND3*, and is associated with a highly angiogenic phenotype and aggressive clinical behavior [4,32,33,34].

By contrast, renal neoplasms with *MITF* gene fusions are exceedingly rare, limited to isolated case reports [35], and there is currently no convincing evidence for recurrent or pathogenic *TFEC* alterations in RCC. Reflecting this molecular landscape, the 2022 WHO classification has moved away from the broad descriptive label “MiT family translocation RCC” and now recognizes two molecularly defined entities: RCC with *TFE3* rearrangement and RCC with *TFEB* alteration [1].

Across these subgroups, a unifying feature is sustained nuclear *TFE3* or *TFEB* activity that is at least partly uncoupled from normal mTORC1-dependent control of subcellular localization, driving transcriptional programs that reshape lysosomal signaling, metabolism, and stress responses [18,23].

### 3.2. Oncogenic Mechanisms and Pathways

MiT-RCC oncogenesis is primarily driven by dysregulated MiT/TFE transcription factors, most commonly through *TFE3* fusion oncoproteins and, in *TFEB*-altered tumors, through either *TFEB* fusions or high-level *TFEB* amplification that reprogram transcription, metabolism, and chromatin architecture. Recent multi-omics studies have begun to map these effects in detail.

**Figure 1:**
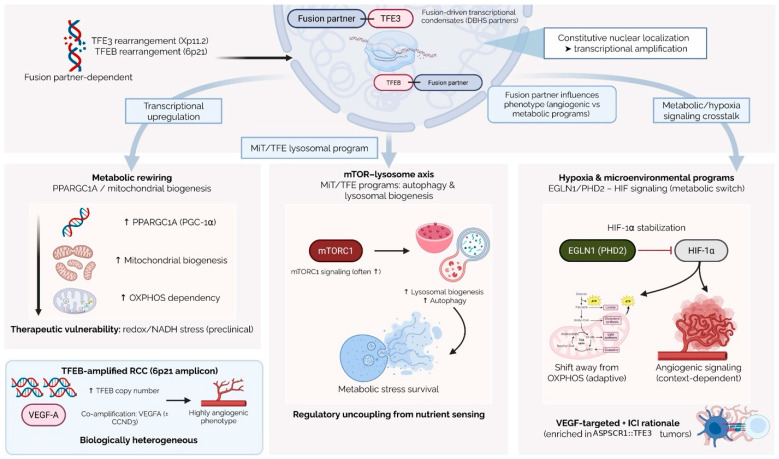
Integrated mechanistic model of MiT-RCC driven by *TFE3* and *TFEB* alterations. In *TFE3*-rearranged RCC, most fusions join a 5′ partner gene to the 3′ portion of *TFE3*, preserving the C-terminal DNA-binding and dimerization domains and promoting constitutive nuclear activity; *TFEB*-rearranged tumors are driven by analogous transcriptional deregulation, whereas *TFEB*-amplified RCC is biologically distinct. These events activate a core MiT/TFE transcriptional program that rewires tumor cell metabolism and stress-adaptation pathways. In fusion-driven translocation RCC (tRCC), *TFE3*/*TFEB* fusions induce upregulation of *PPARGC1A* (PGC-1α), promoting mitochondrial biogenesis and increased dependency on oxidative phosphorylation (OXPHOS), which creates context-specific metabolic vulnerabilities. In parallel, sustained activation of the mTOR-lysosome axis enhances lysosomal biogenesis and autophagy, supporting tumor survival under metabolic stress and reflecting partial uncoupling of MiT/TFE activity from canonical nutrient-sensing control. Crosstalk with hypoxia signaling through the *EGLN1* (PHD2)-HIF-1α axis enables adaptive metabolic switching and, in selected molecular contexts, promotes angiogenic signaling, providing a biological rationale for combined vascular endothelial growth factor (VEGF)-targeted and immune checkpoint inhibitor (ICI) therapies, particularly in angiogenic/stromal-high subtypes such as *ASPSCR1*::*TFE3* tumors. Fusion partner identity shapes transcriptional programs, clinical behavior, and therapeutic responsiveness. A distinct inset highlights *TFEB*-amplified RCC, characterized by increased *TFEB* copy number within the 6p21 amplicon and frequent co-amplification of *VEGFA* (±*CCND3*), driving a highly angiogenic but biologically heterogeneous phenotype that is not obligatorily aligned with the OXPHOS-driven tRCC model. Arrows indicate proposed mechanistic relationships and downstream effects. Created in BioRender. Cotaina Recio, M. (2026) https://BioRender.com/st66q0g (accessed on 12 March 2026).

Proteogenomic and transcriptomic profiling of molecularly confirmed *TFE3*-rearranged RCC has highlighted a prominent oxidative metabolism signature, including elevated oxidative phosphorylation (OXPHOS) and mitochondrial respiratory chain components, together with activation of mTORC1-related signaling [36,37]. In experimental models, *TFE3* fusion oncoproteins can induce transcription of *PPARGC1A* (PGC-1α), promoting mitochondrial biogenesis and shifting tumor cells toward an oxidative metabolic phenotype that may create therapeutic vulnerabilities [38,39]. Consistent with this, genome-wide CRISPR screening has identified *EGLN1* (PHD2) as a candidate metabolic node; *EGLN1* inhibition suppressed tumor growth, diverted metabolism away from OXPHOS, and stabilized HIF-1α in preclinical models [38] (Figure 1).

In keeping with the physiological role of MiT/TFE factors as master regulators of the autophagy-lysosome system, proteogenomic analyses suggest that MiT-RCCs display elevated expression of autophagy and lysosomal gene networks [15,18,36]. This program is hypothesized to help tumor cells withstand metabolic and therapeutic stress by enhancing degradative capacity and nutrient recycling.

An additional oncogenic mechanism involves fusion-driven nuclear condensates. *NONO*::*TFE3* and *SFPQ*::*TFE3* fusion proteins can form liquid-like nuclear condensates that concentrate transcriptional and splicing machinery, remodel chromatin accessibility, and sustain high-level gene expression [40,41,42]. These condensates depend on structural motifs supplied by the fusion partner, particularly coiled-coil domains, and likely contribute to the distinct transcriptional phenotypes observed across different *TFE3* fusion types. Condensate biology therefore represents a potential future therapeutic target.

At the signaling level, mTOR dysregulation is a recurrent theme. Under nutrient-replete conditions, wild-type *TFE3* and *TFEB* are phosphorylated by mTORC1 at the lysosomal surface and retained in the cytoplasm; however, *TFE3* fusion proteins circumvent this control and remain constitutively active in the nucleus, with disrupted 14-3-3 interactions and attenuated relocalization even after pharmacologic mTORC1 inhibition [30]. Additional regulators, such as protein phosphatase 2A (PP2A), can dephosphorylate and activate *TFEB*/*TFE3* independently of mTORC1 inactivation, further amplifying nuclear activity [43]. In murine models of *SFPQ*::*TFE3*-driven tumors, early and persistent mTOR pathway activation has been demonstrated, corroborating mTOR’s role as a downstream effector and key intracellular signaling hub [44].

Unlike clear-cell RCC, which is characterized by frequent point mutations in genes such as *VHL*, *PBRM1*, and *SETD2*, *TFE3*-rearranged RCC tends to have low tumor mutational burden and is enriched for structural and copy-number alterations. Recurrent copy-number gains (notably 17q) and losses (especially 9p21.3 affecting *CDKN2A*/*B*) have been described and correlate with adverse clinicopathologic features in molecular cohorts [45,46,47]. Integrative analyses also implicate activation of *NFE2L2*/*NRF2*-related antioxidant programs in a subset of tumors; this signature has been associated with poorer outcomes on vascular endothelial growth factor receptor (VEGFR)-targeted therapy, while its impact on response to immune checkpoint inhibition remains uncertain [45].

Taken together, *TFE3* fusions function as broad transcriptional amplifiers that simultaneously perturb metabolism, lysosomal function, chromatin organization, and signaling pathways. Reported cooperating alterations are relatively few and appear to converge more on copy-number evolution, cell-cycle dysregulation, oxidative-stress adaptation, and angiogenic programs than on recurrent point mutations. Complementary preclinical models have reinforced these candidate vulnerabilities, including MiT-driven murine systems and fusion-specific functional models that implicate GPNMB, mTOR signaling, and metabolic stress responses [44,48].

### 3.3. TFEB-Rearranged vs. TFEB-Amplified RCC

RCCs with *TFEB* rearrangement and RCCs with *TFEB* amplification are classified together as “RCC with *TFEB* alteration” under the current WHO framework, despite mounting evidence that they represent biologically distinct entities with divergent clinicopathologic and prognostic features [1,49,50]. The t(6;11)(p21;q12) *TFEB* fusion (originally described as “Alpha-*TFEB*” and now recognized as *MALAT1*::*TFEB*) represents the prototypical and most common form of *TFEB*-rearranged RCC [9,31,49]. According to early series, these tumors primarily affect adolescents and young adults, are typically low-grade, kidney-confined, and usually show indolent behavior following nephrectomy [9,31]. Histologically, *TFEB*-rearranged RCC displays a distinctive biphasic pattern consisting of nests of larger clear or eosinophilic epithelioid cells encircling clusters of smaller cells arranged around hyaline basement membrane material, often forming pseudorosettes [51].

Immunophenotypically, these tumors usually exhibit strong expression of melanocytic markers such as cathepsin K, HMB-45, and Melan-A, reflecting *TFEB*-mediated melanocytic differentiation, with variable/limited expression of epithelial markers [50,51,52]. *TFEB* immunohistochemistry is useful as a screening tool, but staining can be variable across *TFEB*-altered tumors (particularly in *TFEB*-amplified RCC) and IHC alone cannot define the underlying mechanism [31,53]. While *MALAT1*::*TFEB* is the canonical fusion, additional less frequent *TFEB* fusion variants (e.g., *ACTB*::*TFEB*, *NEAT1*::*TFEB*) have been described, expanding the molecular spectrum of *TFEB*-rearranged RCC [49,54,55]. Although a small proportion of *TFEB*-rearranged RCCs with additional copy-number alterations and/or higher-grade morphology may exhibit aggressive behavior, contemporary molecularly confirmed cohorts extending into adulthood support that most remain low-stage and clinically indolent. Notably, programmed death-ligand 1 (PD-L1) expression has been reported across *TFEB*-altered RCC cohorts, including *TFEB*-rearranged tumors [52,55,56].

In contrast, *TFEB*-amplified RCC represents a consistently high-grade subtype with aggressive clinical behavior. Typically occurring in middle-aged to older adults (median age ~65 years), these tumors present with high-grade morphology, solid, nested, or pseudopapillary architecture, frequent necrosis, and a resemblance to poorly differentiated clear-cell or papillary RCC [4,32,50,56]. This aggressive phenotype is supported by advanced-stage presentation, lymph-node and distant metastases, and significant disease-specific mortality [32,33]. Immunohistochemically, *TFEB*-amplified tumors may maintain at least focal expression of melanocytic markers (cathepsin K, Melan-A), whereas HMB-45 expression is more variable [50].

The molecular driver of this aggressiveness appears to be gene dosage rather than gene fusion. Unlike the *MALAT1*::*TFEB* fusion, which drives high expression via promoter substitution, *TFEB*-amplified RCC is driven by high-level amplification of the 6p21.1 region. This amplicon typically includes *VEGFA* and often encompasses *CCND3* within the broader 6p21.1 amplified interval [32,50]. Consistent with this, *TFEB*/*VEGFA* amplification is associated with increased *VEGFA* gene copy number and *VEGFA* mRNA expression in *TFEB*-altered RCC, supporting a highly angiogenic phenotype/VEGF pathway dependence. *VEGFA* upregulation can also be observed in a subset of *TFEB*-rearranged tumors (particularly those with additional *TFEB* copy-number gains and more aggressive behavior), suggesting *VEGFA*-driven biology may not be exclusive to the amplified subgroup [57]. Rare tumors with concomitant *TFEB* rearrangement and additional *TFEB* copy-number gains/amplification have also been described and may represent an aggressive subset [58].

Diagnostic distinction is essential. While both subtypes can show nuclear *TFEB* staining, *TFEB*-amplified RCC is defined by increased *TFEB* copy number on FISH (with thresholds varying across series and laboratories), whereas *TFEB*-rearranged RCC requires demonstration of a split signal by break-apart FISH or direct fusion detection by RNA-based assays [4,49,53]. *TRIM63* RNA in situ hybridization (RNA-ISH) can be a useful adjunct, but its sensitivity in *TFEB*-amplified RCC appears variable; therefore, *TRIM63* should be interpreted in context and not used as a stand-alone exclusion test for *TFEB*-altered RCC [59,60]. Given the divergent clinical outcomes, often indolent in younger patients (rearranged) versus aggressive in older patients (amplified), pathology reports should clearly specify the underlying *TFEB* alteration mechanism to guide risk stratification and therapeutic decision-making.

### 3.4. Fusion Partner Heterogeneity and Phenotypic Correlations

Beyond the distinction between *TFE3*- and *TFEB*-driven tumors, a major source of biological heterogeneity in MiT-RCC lies in the identity of the *TFE3* fusion partner. Emerging data suggest that different *TFE3* fusions are associated with distinct clinicopathologic phenotypes, transcriptional programs, and clinical outcomes. In a retrospective cohort of 40 RNA-seq–verified Xp11.2 translocation RCCs, Guo et al. [61] compared four relatively common fusion types (*ASPSCR1*::*TFE3*, *PRCC*::*TFE3*, *SFPQ*::*TFE3*, and *NONO*::*TFE3*) and reported significant differences in progression-free survival across subgroups. In that study, *ASPSCR1*::*TFE3* tumors had significantly shorter PFS compared with other fusion types, whereas overall survival was numerically shorter but not statistically significant, consistent with limited power for OS comparisons. *NONO*::*TFE3* cases showed comparatively favorable outcomes (particularly for PFS), while interpretation for some subtypes was constrained by small numbers and follow-up (notably the *SFPQ*::*TFE3* subgroup).

These findings align with earlier clinicopathologic series indicating that *ASPSCR1*::*TFE3* tumors more often present with adverse stage distribution than *PRCC*::*TFE3* tumors, although outcomes remain strongly stage-dependent and heterogeneity exists within each fusion category [62]. Separate dedicated series also support that many *NONO*::*TFE3* tumors can follow a comparatively indolent clinical course, recognizing that aggressive outliers can still occur [63].

Integrated molecular profiling further supports genotype–phenotype relationships in *TFE3*-rearranged RCC. In a landmark study of untreated primary *TFE3*-rearranged RCC, Sun et al. identified molecular clusters with distinct biological signatures; *ASPSCR1*::*TFE3* tumors mapped predominantly to a cluster characterized by high angiogenesis/stromal enrichment, elevated proliferation signatures, and recurrent 22q loss, and this cluster was associated with the poorest overall survival in that dataset [15]. Complementary proteogenomic analyses also support fusion-partner-associated biological and clinical heterogeneity within MiT-RCC, including differences in disease aggressiveness across fusion subtypes [36].

Mechanistic work provides a structural rationale for some of these differences. *SFPQ* and *NONO* belong to the Drosophila behavior/human splicing (DBHS) family of nuclear RNA-binding proteins. Recent experimental studies show that *SFPQ*::*TFE3* and *NONO*::*TFE3* can form liquid-like nuclear condensates associated with active transcriptional states and altered chromatin accessibility, and that condensate formation depends on coiled-coil domains contributed by the DBHS partner. This condensate-driven transcriptional reprogramming offers a plausible explanation for distinct molecular signatures across DBHS-containing fusions compared with non-DBHS partners [40].

Other fusion partners appear to confer alternative oncogenic properties. The *PRCC*::*TFE3* fusion disrupts the interaction of native *PRCC* with the mitotic checkpoint regulator *MAD2B* and impairs mitotic checkpoint control [64]. Additional, less common partners include epigenetic regulators such as *KAT6A* [65] or *ARID1B* [66], which may point to fusion-specific chromatin biology, although clinically actionable “chromatin dependencies” remain to be established.

Clinically, fusion partner identity has important but not absolute prognostic value and may also prove treatment-relevant. Although *ASPSCR1*::*TFE3* is repeatedly associated with adverse baseline biology in historical series, emerging fusion-annotated retrospective data suggest this subtype may derive particularly strong benefit from modern ICI + VEGFR-TKI combinations [16].

**Figure 2:**
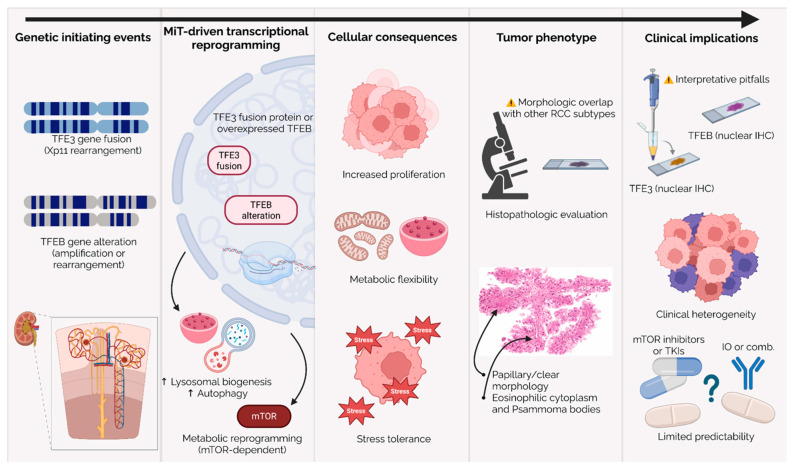
Multistep and multiscale model of MiT family-driven renal cell carcinoma. Schematic overview linking initiating genetic events (*TFE3* gene fusions or *TFEB* gene amplification/rearrangement) to MiT-driven transcriptional reprogramming, cellular consequences, characteristic morphologic patterns, and downstream clinical implications. The diagram integrates molecular alterations with diagnostic pitfalls, clinical heterogeneity, and current therapeutic uncertainty. Arrows indicate progression/relationships between molecular alterations, cellular consequences, tumor phenotype, and clinical implications; colors and icons are schematic and not quantitative. Created in BioRender. Cotaina Recio, M. (2026) https://BioRender.com/8vz33mn (accessed on 12 March 2026).

Taken together, multiple *TFE3* fusion variants, *TFEB*-rearranged tumors, and *TFEB*-amplified RCC comprise a spectrum of molecularly defined MiT-RCC subgroups with distinct transcriptional programs, pathway dependencies, morphologies, and clinical behaviors (Figure 2). As fusion-directed vulnerabilities continue to be uncovered, accurate identification of the underlying fusion event, and where possible the specific partner, will be increasingly important not only for prognosis but also for rational therapeutic stratification. At present, fusion partner identity is clearly associated with morphology, stage distribution, and baseline biology, whereas its role as an independent predictive biomarker for systemic therapy remains preliminary and is based mainly on retrospective fusion-annotated cohorts.

## 4. Pathologic and Diagnostic Approaches

Accurate diagnosis of MiT-RCC requires integrating morphology, IHC, and molecular testing. Suspicion typically arises in settings such as an unusual RCC in a child or young adult, or a renal tumor with clear, papillary, nested/solid, or mixed architecture, often with eosinophilic cytoplasm and frequent psammoma bodies. However, no single morphologic pattern is specific [1,2]. In this context, the initial workup commonly includes IHC for *TFE3* and *TFEB*. Diffuse, strong nuclear staining for either protein supports MiT-RCC, but IHC has important limitations: *TFE3* antibodies can show nonspecific/background nuclear staining in other tumors and even in non-neoplastic cells, and some genetically confirmed *TFE3*-rearranged tumors are only weakly or focally positive. Pre-analytic variables (e.g., prolonged fixation, old blocks, decalcification) and analytic factors (including clone-to-clone variability) further affect assay performance. Accordingly, reliance on *TFE3*/*TFEB* IHC alone is insufficient for definitive diagnosis in many cases, and molecular confirmation is frequently warranted when morphologic or clinical suspicion persists [67]. *TFEB* IHC has analogous pitfalls, particularly in *TFEB*-amplified RCC in which staining may be variable despite high-level copy-number gain, further reinforcing the need for molecular subclassification [4,68].

Some morphologies can nevertheless raise suspicion for specific rearrangements—for example, papillary architecture in *PRCC*::*TFE3* RCC, glomeruloid/biphasic *TFEB*-like architecture in *SFPQ*::*TFE3* tumors, and multilocular cystic morphology in *MED15*::*TFE3* RCC. These patterns can also help tailor the initial immunohistochemical panel when more common mimics are in the differential diagnosis; for example, carbonic anhydrase IX may be informative in clear cell-like tumors and CK7 in papillary-appearing tumors before proceeding to MiT-RCC-directed markers and molecular confirmation [69].

In addition to *TFE3* and *TFEB*, cathepsin K has historically been used as a supportive immunohistochemical marker for MiT-RCC, reflecting downstream activation of MiT/TFE transcriptional programs. Biologically, cathepsin K is a MiT-family–regulated gene, providing a mechanistic rationale for its use as a “readout” of MiT/TFE pathway activation [70]. In practice, however, cathepsin K expression is not uniform across MiT-RCC: it is consistently expressed in *TFEB*-rearranged t(6;11) RCC and is common in *PRCC*::*TFE3* RCC, but can be absent in *ASPSCR1*::*TFE3* RCC, underscoring that cathepsin K negativity does not exclude MiT-RCC [71]. Moreover, specificity is limited because cathepsin K expression is also observed across a broader range of human neoplasms beyond MiT-RCC, including melanoma, granular cell tumors, and alveolar soft part sarcoma [72]. Consequently, cathepsin K should be regarded as an adjunctive marker that can raise suspicion in the appropriate morphologic context, but does not establish the diagnosis on its own.

Importantly, strong nuclear *TFE3* immunoreactivity is not fully specific for a *TFE3* gene fusion. Increased *TFE3* copy number (including focal amplification and X-chromosome polysomy) can result in *TFE3* overexpression detectable by IHC in the absence of a rearrangement, representing a defined molecular mechanism for *TFE3* IHC positivity without translocation. This phenomenon is particularly relevant in adult high-grade RCCs and represents an important source of false-positive IHC results [73]. Therefore, molecular confirmation is recommended whenever *TFE3* IHC is positive in an atypical clinical or morphologic setting, and whenever therapeutic or prognostic decisions hinge on establishing a MiT-RCC diagnosis.

To address these challenges, GPNMB (glycoprotein NMB) has emerged as a sensitive screening immunohistochemical surrogate for MiT-RCC. Mechanistically, GPNMB is transcriptionally activated in *TFE3* fusion–driven RCC and has been validated as a robust IHC marker in fusion-confirmed cohorts [74]. In a large reference-laboratory series evaluating 3606 renal tumors for *TFE3* and *TFEB* alterations, diffuse GPNMB expression was reported in 92% of *TFE3*-rearranged RCCs and 100% of *TFEB*-rearranged and *TFEB*-amplified RCCs, supporting its value as a screening marker in equivocal cases [68]. Because GPNMB expression can occur in other settings, it should be interpreted as supportive and used to triage tumors for definitive molecular testing rather than as a replacement for confirmatory assays.

For confirmatory testing, break-apart FISH remains widely used and is commonly treated as a reference standard for establishing *TFE3* or *TFEB* rearrangement status in routine practice [53]. However, FISH can yield false-negative or equivocal results, especially with complex or intrachromosomal events in which the separation between probes is subtle. In addition, break-apart FISH may fail to show a classic “split” pattern for rearrangements involving partner genes located very close to *TFE3* on Xp11.2 (e.g., *NONO*), creating a recognized diagnostic blind spot if FISH results are interpreted in isolation [53]. Cryptic rearrangements can also evade routine probe interpretation; for example, *RBM10*::*TFE3* fusions arising from small paracentric Xp11.2 inversions may be “FISH-concealed,” requiring heightened awareness and/or alternative molecular approaches for detection [75,76]. Finally, *TFEB*-amplified RCC will not show a rearrangement split pattern on *TFEB* break-apart FISH because the gene is not translocated; instead, copy-number assessment is required (e.g., *TFEB* signal enumeration on FISH, array comparative genomic hybridization, or NGS-based copy-number analysis) [4,32]. Consistent with this, contemporary diagnostic approaches increasingly emphasize integrating IHC surrogates (such as GPNMB) with FISH and sequencing-based methods in challenging cases [68].

To improve detection in diagnostically difficult tumors, novel ancillary assays have been developed. One major advance is *TRIM63* (MuRF1) mRNA in situ hybridization (RNA-ISH), leveraging *TRIM63* overexpression as a downstream transcriptional signal in MiT-RCC. In the original report by Wang et al. [59] (177 RCC cases including 31 cytogenetically confirmed MiT-RCCs), *TRIM63* RNA-ISH showed high-level signal in 89% of confirmed cases. Using an H-score cutoff of 168, the assay achieved 90% sensitivity and 100% specificity in that cohort, with an area under the curve of 0.985. Importantly, *TRIM63* RNA-ISH was strongly positive in FISH-negative tumors harboring cryptic rearrangements, demonstrating its ability to flag cases that can elude conventional break-apart FISH.

External validation has supported the assay’s utility while highlighting limitations. In an independent cohort of 331 renal tumors, *TRIM63* RNA-ISH was positive in 80% of *TFE3*-translocation RCCs and 33% of *TFEB*-amplified RCCs. Using the H-score cutoff of 168, overall sensitivity was 72% and specificity 96%, with a high negative predictive value of 98%. However, *TRIM63* positivity was enriched in select eosinophilic renal neoplasms, reinforcing that results should be interpreted in morphologic and molecular context rather than as a stand-alone classifier [60]. Sensitivity is lower for *TFEB*-driven tumors than for *TFE3*-driven tumors, so a negative *TRIM63* result does not exclude MiT-RCC, particularly in *TFEB*-amplified cases.

A recent study further evaluated *TRIM63* in clinically relevant “discordant” scenarios. Among 20 RCCs that were *TRIM63*-positive but *TFE3*/*TFEB* FISH-negative or equivocal, additional sequencing confirmed *TFE3* or *TFEB* alterations in 70% (14/20), including frequent *RBM10*::*TFE3* inversions, underscoring that initial FISH can miss a meaningful subset of bona fide MiT-RCC and supporting *TRIM63* RNA-ISH as an adjunct in unclassified or diagnostically difficult renal tumors [77].

Increasingly, comprehensive molecular profiling is also used for definitive diagnosis and subclassification. RNA-based fusion testing (e.g., targeted RNA sequencing or anchored multiplex PCR on formalin-fixed, paraffin-embedded tissue) can directly detect *TFE3*/*TFEB* fusion transcripts and identify the fusion partner, enabling both confirmation and clinically relevant subclassification in a single assay [78]. This is particularly valuable in cases with atypical FISH patterns or “FISH-concealed” fusions (e.g., *RBM10*::*TFE3*) [76]. Broad DNA/RNA NGS platforms can additionally provide copy-number profiling, which is useful for recognizing high-level 6p21 gains consistent with *TFEB* amplification when *TFEB* IHC is positive but rearrangement testing is negative [4,32].

**Figure 3:**
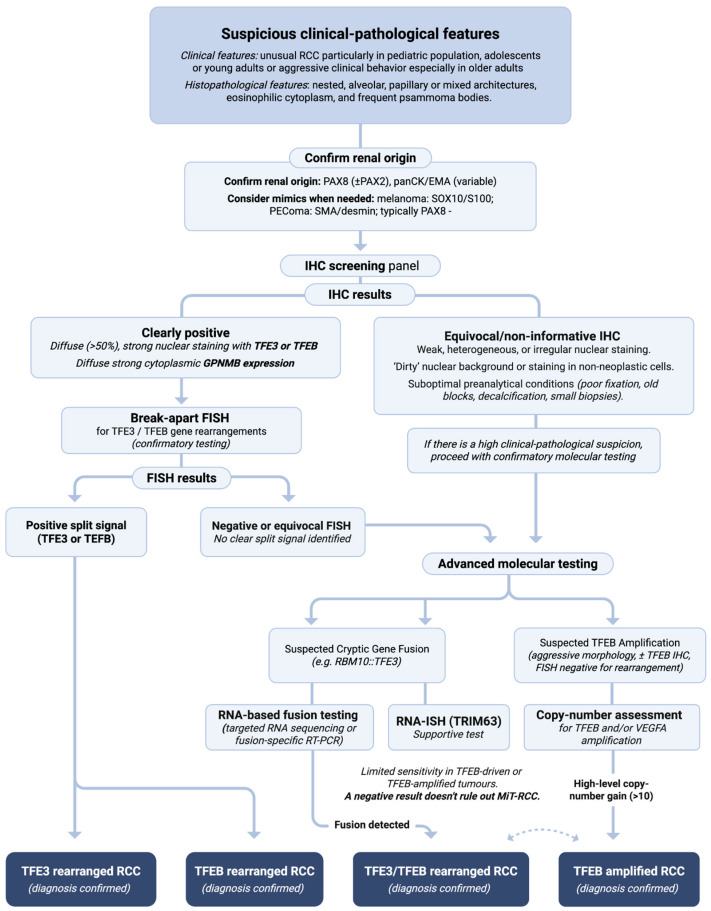
Suggested diagnostic workflow for suspected MiT family renal cell carcinoma (MiT-RCC). The algorithm integrates clinical-pathologic suspicion, confirmation of renal origin, an immunohistochemistry (IHC) screening panel (*TFE3*, *TFEB*, and GPNMB), confirmatory break-apart fluorescence in situ hybridization (FISH) for rearrangements where appropriate, and advanced molecular testing (RNA-based fusion assays, *TRIM63* RNA in situ hybridization (RNA-ISH), and copy-number assessment for *TFEB*/*VEGFA* amplification) in equivocal cases. Abbreviations: EMA, epithelial membrane antigen; FISH, fluorescence in situ hybridization; GPNMB, glycoprotein NMB; IHC, immunohistochemistry; panCK, pancytokeratin; PAX2, paired box gene 2; PAX8, paired box gene 8; PEComa, perivascular epithelioid cell neoplasm; RNA-ISH, RNA in situ hybridization; RT-PCR, reverse transcription polymerase chain reaction; SMA, smooth muscle actin; S100, S100 protein; SOX10, SRY-box transcription factor 10; *VEGFA*, vascular endothelial growth factor A. Created in BioRender. Cotaina Recio, M. (2026) https://BioRender.com/6q7tzvy (accessed on 12 March 2026).

A practical diagnostic workflow for suspected MiT-RCC can proceed in stages: morphologic assessment with an IHC screening panel (*TFE3*, *TFEB*, and GPNMB), followed by *TFE3*/*TFEB* rearrangement testing (often break-apart FISH) when suspicion persists, then *TRIM63* RNA-ISH and/or RNA-based fusion testing when FISH is negative/equivocal but clinicopathologic suspicion remains high, and copy-number assessment for *TFEB* amplification (±*VEGFA* co-gain, depending on assay design) in aggressive *TFEB*-positive, rearrangement-negative tumors (Figure 3).

This multimodal approach maximizes both sensitivity and specificity and reduces the risk of missing cryptic MiT fusions. Early and accurate identification of MiT-RCC is clinically important, as these tumors may prompt different management considerations than other RCC subtypes and may qualify patients for fusion-specific clinical trials.

Finally, beyond fusion detection, integrative transcriptomic and proteogenomic studies continue to refine biologic subclassification within MiT-RCC and may eventually generate ancillary molecular classifiers that help distinguish MiT-RCC from morphologic mimics in particularly challenging cases, although such approaches remain investigational and are not yet incorporated into routine diagnostic practice [36,45]. Emerging artificial-intelligence tools for digital pathology are also being explored to triage cases for molecular testing, but remain investigational at present.

## 5. Therapeutic Evidence and Current Management

The management of advanced (metastatic) MiT-RCC remains a clinical challenge given the rarity of this disease and the absence of dedicated phase III trials [45,79,80]. Treatment strategies have largely been extrapolated from clear-cell RCC, as no approved therapies exist specifically for this histologic subtype [45,81]. Historically, outcomes with conventional targeted therapies (VEGF TKIs or mTOR inhibitors used as monotherapy) have been poor, with reported objective response rates of only 10–17% and median progression-free survival of 3–8 months [80,81,82]. Over the last few years, however, prospective non-clear-cell RCC studies and large multi-institutional retrospective series have begun to clarify more effective systemic strategies in both adult and pediatric MiT-RCC. Below, we summarize the evidence for VEGF-targeted tyrosine kinase inhibitors (TKIs), immune checkpoint inhibitors (ICIs), combination regimens, mTOR-pathway inhibitors, and newer experimental strategies, and we discuss current management recommendations. Table 1 provides an overview of key clinical studies in MiT-RCC and is intended to contextualize evidence strength by showing study design, line of therapy, molecular confirmation, and key limitations. Unless otherwise specified, the systemic-therapy evidence summarized below largely reflects fusion-driven MiT-RCC (tRCC), because *TFEB*-amplified RCC is rare and underrepresented in prospective and retrospective treatment series. Across studies, interpretation is constrained by small sample sizes, retrospective designs with heterogeneous lines/regimens, and frequent absence of uniform molecular confirmation and/or fusion-partner annotation in older cohorts. Except for a few small randomized or prospective subsets, most comparative treatment signals remain retrospective and should be interpreted as hypothesis-generating rather than definitive.

### 5.1. TFEB-Amplified RCC: Systemic Therapy Considerations

*TFEB*-amplified RCC warrants separate discussion given its distinct biology and aggressive clinical behavior. This subtype is rarely represented in prospective non-clear-cell RCC trials and is often not separately analyzed in retrospective translocation RCC series, so systemic therapy recommendations are extrapolated and should be framed as hypothesis-generating.

Biologically, high-level *TFEB* amplification typically occurs within a 6p21.1 amplicon that includes *VEGFA* (and frequently encompasses *CCND3*), providing a rationale for VEGFR-targeted therapy in eligible patients [32]. Limited clinical evidence, primarily case reports and small series, describes disease control with VEGFR-targeted TKIs in *TFEB* and *VEGFA* co-amplified tumors [32,34]. However, in a recent series with detailed treatment data, all three patients treated with single-agent VEGFR-TKI experienced progression, whereas those receiving PD-1 inhibitor–based therapy (*n* = 3) achieved disease control [56].

Membranous PD-L1 expression has been reported frequently in *TFEB*-amplified cohorts [33,56], providing additional biological rationale for immune checkpoint inhibition. Although these preliminary observations are encouraging, larger studies are needed to confirm efficacy, define optimal regimens, and determine whether *TFEB*-amplified RCC responds similarly to fusion-driven tRCC.

At present, no evidence-based systemic standard of care exists for *TFEB*-amplified RCC. For fit patients, an ICI plus VEGFR-TKI combination may be considered on the basis of biologic plausibility and limited case-based experience, but subtype-specific comparative data are lacking and clinical trial enrollment should be prioritized whenever feasible [83,84].

### 5.2. Immune Checkpoint Inhibitor (ICI)-Based Strategies

#### 5.2.1. ICI + VEGF/VEGFR-Targeted Therapy (ICI + TKI Combinations)

For fit patients with metastatic fusion-driven MiT-RCC (tRCC, defined by *TFE3* or *TFEB* rearrangement), combination regimens pairing an anti-PD-1/PD-L1 immune checkpoint inhibitor with VEGF/VEGFR-targeted therapy are increasingly used and are supported by converging prospective subsets and multi-institutional retrospective series. A plausible biologic explanation is that at least a subset of tRCC, particularly angiogenic/stromal-high tumors, may be more susceptible to VEGF-sensitized immunotherapy. These data suggest that, although translocation RCC has often been considered less immunogenic than clear-cell RCC, clinically meaningful responses can occur when checkpoint blockade is combined with anti-angiogenic agents. Most available systemic-therapy data labeled “MiT-RCC” or “tRCC” derive from tumors with *TFE3* or *TFEB* rearrangements rather than *TFEB* amplification, and the discussion in this section reflects that evidence base.

The single-arm phase II KEYNOTE-B61 trial evaluated pembrolizumab plus lenvatinib as first-line therapy in 158 patients with advanced non-clear-cell RCC [85,86]. A translocation RCC subgroup was included (*n* = 6, investigator-assessed), representing one of the few prospectively enrolled tRCC cohorts treated with first-line ICI + TKI. Across the overall non-clear-cell population, the primary analysis reported a confirmed objective response rate (ORR) of 49%, and updated results with longer follow-up (median 22.8 months) showed a confirmed ORR of 51% (including 8.2% complete responses), median progression-free survival (PFS) of 17.9 months, and median overall survival (OS) not reached. Within KEYNOTE-B61, the translocation RCC subgroup achieved an ORR of 66.7% (4 of 6 patients) and a disease control rate of 83.3% (5 of 6). These results should be interpreted as signal-seeking given the very small translocation subset and the lack of fusion-partner annotation and molecular subclassification (*TFE3* versus *TFEB* rearrangement), but they provide important prospective proof-of-concept that PD-1 blockade combined with VEGFR-targeted therapy is active in histologically defined translocation RCC.

A complementary prospective signal comes from the single-arm phase II study of cabozantinib plus nivolumab in non-clear-cell RCC. In cohort 1, which included patients with papillary, unclassified, and translocation-associated RCC (*n* = 40), the combination achieved an ORR of 47.5%, with a median PFS of 12.5 months and median OS of 28 months [87]. With extended follow-up (median 34 months), the efficacy signal remained robust, with an ORR of 48%, median PFS of 13 months, and median OS of 28 months, and a safety profile consistent with prior reports [88]. Although limited to only two patients with tRCC, one of whom achieved a confirmed partial response, these results align with the broader efficacy of cabozantinib-based ICI combinations and reinforce the concept that aggressive non-clear-cell histologies, including tRCC, are amenable to ICI plus multitarget TKI regimens.

Randomized comparator evidence supporting checkpoint blockade combined with VEGF inhibition in TFE-fusion RCC comes from an exploratory transcriptomic analysis of the phase III IMmotion151 trial (atezolizumab plus bevacizumab versus sunitinib). Rare tumors harboring *TFE3* or *TFEB* fusions identified by RNA sequencing (*n* = 15; 12 *TFE3* and 3 *TFEB*) had markedly improved progression-free survival with atezolizumab plus bevacizumab compared with sunitinib (median PFS 15.8 vs. 3.5 months; hazard ratio (HR) 0.13; *p* = 0.004) [89]. Despite the limited sample size and the fact that these patients were not prospectively enrolled as translocation RCC, this analysis provides rare randomized evidence consistent with the higher activity of checkpoint blockade sensitized by anti-angiogenic therapy observed in dedicated tRCC cohorts.

Consistent with these prospective signals, MiT-RCC-focused retrospective cohorts with molecular confirmation have also reported higher response rates with ICI + VEGF-TKI combinations than with ICI monotherapy or dual ICI [80,90]. However, these apparent differences derive mainly from small retrospective series and limited prospective subsets rather than definitive subtype-specific comparative trials.

Beyond aggregate outcomes, emerging data suggest that molecular heterogeneity within *TFE3*-rearranged RCC may influence treatment response. In a retrospective cohort of metastatic *TFE3*-rearranged RCC treated with first-line ICI-based combination therapy with known fusion partner, *ASPSCR1*::*TFE3* tumors achieved an ORR of 62.5% (5/8) with median PFS not reached, compared with an ORR of 10% (1/10) and median PFS of 6.5 months in non-*ASPSCR1* fusions; these findings are hypothesis-generating and are discussed in greater detail in Section 6 [16].

Looking forward, randomized evidence dedicated to non-clear-cell RCC is expanding. The phase III STELLAR-304 trial (NCT05678673) is evaluating zanzalintinib (XL092) plus nivolumab versus sunitinib as first-line therapy in advanced non-clear-cell RCC, although whether it will accrue a sufficiently large translocation RCC subset for definitive conclusions remains to be seen [91]. Earlier-phase data are also emerging from STELLAR-002 (NCT05176483), a phase 1b dose-escalation and cohort-expansion study of zanzalintinib alone and in combination with immuno-oncology agents that includes RCC expansion cohorts. Until such data mature, ICI + TKI combinations remain the preferred first-line approach for fit patients with metastatic fusion-driven MiT-RCC based on available prospective and retrospective evidence.

#### 5.2.2. ICI Monotherapy and Dual Checkpoint Blockade: Current Evidence and Limitations

A central clinical question is whether fusion-driven MiT-RCC (tRCC) can be treated with immune checkpoint inhibitors alone either as monotherapy or as dual checkpoint blockade (cytotoxic T-lymphocyte-associated protein 4 [CTLA-4] plus PD-1), thereby sparing patients the toxicity of a TKI. No randomized trial has directly compared ICI monotherapy versus dual ICI specifically in tRCC, and available evidence derives from small retrospective series and non-clear-cell RCC trials that include only limited numbers of translocation cases. In clear-cell RCC, dual checkpoint blockade with nivolumab plus ipilimumab can yield durable complete responses in a subset of patients [92,93], but this pattern has not been reproduced at a cohort level in translocation RCC.

Across translocation RCC series, both ICI monotherapy and dual ICI generally show modest activity, whereas ICI plus TKI combinations achieve consistently higher response rates and improved disease control, making them the preferred systemic approach in eligible patients with metastatic translocation RCC.

The only randomized, translocation-RCC-dedicated prospective dataset to date comes from the phase II AREN1721 trial, a Children’s Oncology Group (COG)-led study in unresectable or metastatic *TFE3*- or *TFEB*-rearranged RCC that enrolled both pediatric and adult patients [94]. The study was closed early for poor accrual after enrolling 15 patients (13 eligible), with a median age of 16 years and a predominance of pediatric and adolescent and young adult (AYA) cases. Patients were randomized to nivolumab plus axitinib, axitinib alone, or nivolumab alone. Despite its small size, the randomized comparison between combination therapy and nivolumab monotherapy was striking. Among six patients assigned to nivolumab plus axitinib, 33% achieved a partial response and none had primary progression, whereas in the nivolumab-alone arm (*n* = 5) there were no objective responses and early progression was common. Median PFS improved from 1.8 months with nivolumab to 10.5 months with the combination (one-sided *p* = 0.0004), and OS was also significantly superior with the combination (*p* = 0.003). These *p*-values, while nominally significant, should be interpreted cautiously given the extremely small randomized groups (*n* = 6 for nivolumab plus axitinib and *n* = 5 for nivolumab monotherapy), early trial closure, and inherently unstable point estimates. In addition, early closure and the pediatric/AYA-skewed enrollment limit generalizability to typical adult metastatic tRCC and make arm-to-arm estimates particularly unstable. Although the trial is underpowered, heavily pediatric, and the axitinib-only arm was too small to clarify the incremental value of adding PD-1 blockade, AREN1721 provides randomized evidence that single-agent nivolumab has very limited activity in translocation RCC, whereas the nivolumab plus axitinib doublet can induce meaningful and durable disease control. As these findings are currently available only in abstract form, they should be considered preliminary pending peer-reviewed publication.

A 2023 multicenter retrospective study evaluated ICI-based regimens in 29 adults with advanced *TFE3*- or *TFEB*-rearranged RCC, all confirmed by FISH [80]. Dual ICI, most often nivolumab plus ipilimumab, was used in 18 patients, and ICI combined with VEGF/VEGFR-targeted therapy was used in 11. Seventeen patients (59%) received an ICI combination in the first-line setting. In the ICI + VEGF/VEGFR-targeted therapy group, most commonly axitinib- or cabozantinib-based regimens, with a minority receiving atezolizumab plus bevacizumab, the ORR was 36% with a median PFS of 5.4 months. In contrast, dual ICI produced an ORR of 5.6% (1 of 18 patients) and a median PFS of 2.8 months. These data support higher upfront response activity and longer disease control with VEGF/VEGFR-sensitized ICI regimens than with dual checkpoint blockade in adult tRCC, while recognizing the limitations of retrospective design, small sample size, and heterogeneity in regimens and lines of therapy.

Earlier reports are concordant. In an international multicenter retrospective series of 24 patients with metastatic *TFE3*- or *TFEB*-rearranged RCC (tRCC), immune checkpoint inhibitors were administered primarily in second-line or later settings (first ICI exposure), predominantly nivolumab or ipilimumab monotherapy, with a minority receiving ICI-based combinations in later lines. First ICI exposure was associated with an ORR of 16.7% and a median PFS of 2.5 months; 12.5% of patients achieved stable disease [95]. Durable responses were documented in a small subset but clearly represented the exception rather than the rule.

A 2025 multi-institutional retrospective analysis focused specifically on combination immunotherapy in 22 patients with metastatic *TFE3*-rearranged RCC confirmed by FISH [90]. ICI plus VEGF-TKI combinations (*n* = 14) achieved a higher ORR than ICI plus ICI (54% versus 14%) and a longer median time to treatment failure (6.2 versus 1.2 months). Median OS was numerically longer in the dual-ICI cohort (36.7 versus 15.6 months), but this difference was not statistically significant. The paradoxical finding of longer median OS in the dual-ICI group despite much lower response rates likely reflects a combination of small sample size, differences in baseline risk, subsequent lines of therapy, and the recognized “tail of the curve” phenomenon with checkpoint blockade, whereby the minority of patients who respond to dual ICI may achieve exceptionally durable disease control. This observation should not be interpreted as evidence of superior efficacy for dual ICI over ICI plus TKI combinations. Overall, these data indicate that while dual ICI is feasible and can occasionally produce durable benefit, its upfront response activity in *TFE3*-rearranged RCC is limited, whereas ICI plus TKI induces objective responses in a clinically meaningful fraction of patients.

Randomized non-clear-cell RCC data provide additional context. The randomized phase II SUNNIFORECAST trial compared ipilimumab plus nivolumab with investigator’s-choice standard-of-care therapy, which consisted predominantly of VEGFR-TKIs with a minority of ICI plus TKI regimens, in previously untreated non-clear-cell RCC [96]. Ipilimumab plus nivolumab improved the 12-month OS rate (78% versus 68%; primary endpoint; *p* = 0.026), increased ORR (32.8% versus 19.3%), and produced similar PFS (hazard ratio 0.99); treatment discontinuation for toxicity occurred in 17% versus 9%. Central review identified a small subgroup with translocation RCC (7.6% of the ipilimumab plus nivolumab arm and 3.3% of the standard-of-care arm), but outcomes were not reported separately for this subset and the standard-of-care arm combined TKIs with and without ICI. SUNNIFORECAST therefore supports the feasibility and overall activity of dual ICI in broad non-clear-cell RCC but does not provide translocation RCC-specific efficacy data and does not alter the preference for ICI plus TKI when translocation RCC is confirmed.

Taken together, adult and pediatric data suggest that fusion-driven MiT-RCC, especially *TFE3*- and *TFEB*-rearranged tumors, is not reliably immune responsive to checkpoint blockade alone. Integrative clinical-molecular analyses provide additional context: Bakouny et al. demonstrated that translocation RCC harbors a heightened *NRF2*-driven antioxidant response associated with resistance to targeted therapy and reported worse outcomes on VEGFR-TKI therapy than on ICI within translocation RCC cohorts [45]. While this does not directly establish mechanistic synergy, the higher response activity observed with ICI + VEGF/VEGFR-targeted combinations than with ICI alone across small series is consistent with a contributory role for VEGF/VEGFR blockade in enabling effective checkpoint blockade in at least a subset of tRCC. In current practice, ICI plus TKI combinations are therefore preferred over ICI monotherapy or dual ICI as first-line systemic therapy for metastatic *TFE3*- or *TFEB*-rearranged RCC in eligible patients, recognizing that most evidence is retrospective and that translocation RCC subset sizes in prospective trials remain small. Single-agent or dual-ICI approaches are generally reserved for patients who cannot receive VEGF-directed TKIs or for later-line settings, with the expectation of lower response rates and shorter disease control, while acknowledging that rare exceptional responders can occur. Prospective validation, particularly in fusion-partner-annotated cohorts, remains needed.

### 5.3. VEGF/VEGFR Tyrosine Kinase Inhibitor (TKI) Monotherapy

#### 5.3.1. Cabozantinib

Cabozantinib has one of the strongest evidence bases in metastatic fusion-driven MiT-RCC. In a multicenter retrospective analysis of 52 patients with metastatic MiT-RCC treated with cabozantinib (many previously exposed to VEGFR-targeted therapy and/or immune checkpoint inhibitors), the objective response rate was 17.3% and stable disease was observed in 50.0%, with a median progression-free survival of 6.8 months and median overall survival of 18.3 months [81]. Durable clinical benefit (typically defined as CR/PR/SD lasting ≥ 6 months) was observed in approximately 46% of patients, supporting cabozantinib as a key option, particularly when not used in earlier ICI-based combinations [81].

#### 5.3.2. Classical VEGF/VEGFR-Targeted TKIs

Prior to the immune checkpoint inhibitor era, first-generation VEGFR TKIs (e.g., sunitinib, sorafenib, pazopanib, axitinib) were commonly used in metastatic fusion-driven MiT-RCC, with generally modest outcomes. In a multicenter retrospective tRCC cohort, first-line VEGFR-TKI therapy (predominantly sunitinib) yielded an objective response rate of 10.5% and a median progression-free survival of 3.0 months [95]. In a separate single-center series of 45 patients with metastatic Xp11.2 translocation RCC, median progression-free survival was 7.4 months and median overall survival was 17.9 months with VEGFR-TKI therapy; in that report, two patients who received first-line VEGFR-TKI plus ICI achieved prolonged progression-free survival (>16.6 and >25.6 months), supporting ongoing interest in combination strategies while recognizing the very small numbers [97].

In contemporary practice, classical VEGFR-TKI monotherapy has largely been supplanted by ICI-based combinations and/or cabozantinib-containing regimens in eligible patients. Nevertheless, VEGFR TKIs remain reasonable options when immune checkpoint inhibitors are contraindicated (e.g., active autoimmune disease, solid-organ transplant) or as later-line palliative therapy after progression on immune-based combinations. Integrative analyses have described an *NRF2*/oxidative-stress response signature in tRCC (predominantly *TFE3*-rearranged, with smaller *TFEB*/*MITF* subsets) associated with poorer outcomes on VEGFR-TKI therapy, which may contribute to the limited durability of VEGFR-TKI monotherapy in this subtype [45].

### 5.4. mTOR Pathway Inhibitors

The rationale for targeting the mTOR pathway in MiT-RCC is supported by preclinical data. *TFE3* fusion proteins can transcriptionally upregulate *IRS1* and activate PI3K/AKT/mTOR signaling, as shown by ChIP-seq and functional studies in *TFE3*-rearranged RCC models [98]. In parallel, experimental data indicate that *TFE3* fusion proteins may escape normal mTORC1-dependent cytoplasmic sequestration, with reduced 14-3-3 interactions and persistent nuclear localization even after pharmacologic mTORC1 inhibition [30]. This biology may help explain why mTOR inhibition alone has not consistently translated into robust clinical activity in fusion-driven MiT-RCC, despite a mechanistic rationale.

Clinically, evidence for single-agent mTOR inhibitors in MiT-RCC is limited and largely extrapolated from broader non-clear-cell RCC populations. In a Memorial Sloan Kettering retrospective series of metastatic non-clear-cell RCC treated with temsirolimus or everolimus (*n* = 85), median progression-free survival was 2.9 months and the objective response rate was 7%; importantly, no tumor shrinkage was observed among the small translocation-associated RCC subset (*n* = 3) [99].

Combination strategies therefore remain of interest. In a randomized open-label, three-arm phase II trial in previously treated metastatic clear-cell RCC, lenvatinib plus everolimus improved progression-free survival compared with everolimus alone (median 14.6 vs. 5.5 months; hazard ratio 0.40), supporting the general principle that combining a VEGFR-pathway TKI with mTOR inhibition can improve efficacy over mTOR inhibition alone in RCC. However, MiT-RCC–specific data are lacking, so use in MiT-RCC remains an extrapolation and is generally reserved for later-line practice when clinical trial access is limited [100].

Preclinical models also support combination strategies: *TFE3*-fusion cell lines and xenografts show sensitivity to dual PI3K/mTOR pathway suppression [98], and cabozantinib combined with the mTORC1/2 inhibitor sapanisertib induced tumor regression in patient-derived RCC xenografts, including models derived from tumors that had progressed on approved VEGFR-TKI plus immunotherapy combinations [101]. While not MiT-RCC–specific, these data support continued investigation of rational VEGFR/mTOR co-targeting strategies.

### 5.5. HIF-2α Inhibitors

Belzutifan is an oral HIF-2α inhibitor approved for von Hippel–Lindau (*VHL*) disease–associated tumors requiring systemic therapy, including RCC [102], and it has demonstrated clinical activity in previously treated advanced clear-cell RCC in phase III testing (LITESPARK-005; belzutifan versus everolimus) [103,104]. However, MiT-RCC is defined by *TFE3*/*TFEB* alterations rather than canonical *VHL*/HIF biology [1], and MiT-RCC–specific efficacy data for belzutifan have not been reported (no dedicated series and no prospectively analyzed translocation-RCC subsets). Therefore, belzutifan should be considered investigational in this setting and ideally used in the context of a clinical trial.

### 5.6. Epigenetic and Novel Targeted Approaches

Several investigational strategies are being explored to exploit the distinctive biology of MiT/TFE-driven tumors. GPNMB is strongly upregulated in *TFE3*-fusion RCC and represents a potentially targetable surface antigen, although clinical validation and MiT-RCC-specific trials are lacking [74]. Preclinical drug-screening efforts have also nominated candidate agents, including transcriptional inhibitors such as mithramycin A, that warrant further study in appropriate MiT-RCC models [105].

Recent work has highlighted metabolic dependencies in fusion-driven MiT-RCC, including transcriptional programs that favor oxidative metabolism/oxidative phosphorylation relative to the glycolytic bias typical of clear-cell RCC. In preclinical models, perturbation of these pathways, including targeting *EGLN1*/HIF-axis signaling, has been proposed as one potential strategy [38]. In addition, rare *TFE3* fusion partners involve chromatin-regulatory genes such as *KAT6A* (a histone acetyltransferase) and *ARID1B* (a SWI/SNF complex component), raising the hypothesis that chromatin biology may differ across fusion subtypes. That being said, clinically actionable epigenetic dependencies remain unproven at present [65,66]. It should be emphasized that these approaches remain preclinical or early investigational, and prospective clinical trials will be required before they can be recommended in routine management of MiT-RCC.

### 5.7. Pediatric and Adult Treatment Nuances

Pediatric and adolescent patients with *TFE3*- or *TFEB*-rearranged RCC pose special considerations, but systemic therapy recommendations are typically extrapolated from adult RCC guidelines because prospective pediatric data are scarce [106]. Surgery remains the cornerstone for localized disease, and available pediatric reviews/series suggest that conventional cytotoxic chemotherapy has little or no established role in metastatic RCC [107]. In the pre-immune checkpoint inhibitor era, the Juvenile RCC Network reported objective responses to VEGFR-targeted therapy in metastatic Xp11.2/*TFE3* translocation RCC (age range 2–45 years; median 34 years), including partial responses to sunitinib and a longer median progression-free survival with first-line sunitinib than with cytokine therapy [108].

Consistent with these observations, in a single-institution pediatric series (median age 15 years) in which all patients with stage IV disease had translocation morphology RCC, anti-angiogenic therapy was associated with the most consistent benefit; the longest mean time to progression was reported with axitinib (7.8 months) and sunitinib (4.7 months) [106]. Other VEGFR TKIs (e.g., pazopanib) and cabozantinib have been used in individual pediatric RCC cases, although molecular subtype reporting is variable [106,109]. Randomized data from AREN1721 (COG; abstract only) suggest that immune checkpoint inhibitor monotherapy has very limited activity in pediatric/AYA translocation RCC, whereas nivolumab plus axitinib can induce objective responses and meaningful disease control [94].

Molecular subtype remains clinically relevant across ages. *TFEB*-rearranged t(6;11) RCC is typically diagnosed in younger patients and many reported cases have been localized and indolent after surgical resection, although aggressive and metastatic behavior has been described, supporting careful long-term follow-up [9,110]. For *TFE3*-rearranged RCC, fusion partner influences phenotype: in multi-omic cohorts, *ASPSCR1*::*TFE3* tumors have been enriched for high-grade features and lymph node/distant metastases and have had inferior outcomes compared with other *TFE3* fusions [15]. Treatment decisions in children should balance potential benefit against long-term toxicity, and multidisciplinary management and trial/registry enrollment remain particularly important in this rare setting.

## 6. Outcomes and Prognosis

MiT-RCC encompasses molecularly defined renal cancers with heterogeneous natural histories. Most historical outcome data, particularly from the VEGFR-TKI era, derive from fusion-driven translocation RCC (tRCC; *TFE3*- or *TFEB*-rearranged) rather than *TFEB*-amplified RCC. In early metastatic adult series treated predominantly with VEGFR-targeted monotherapy, outcomes were generally poor (e.g., median overall survival ~14 months), although estimates varied across small cohorts and were strongly stage-dependent [95,111]. Contemporary molecularly annotated cohorts and multi-omic studies further underscore substantial biologic and clinical heterogeneity within tRCC [36].

As detailed in Section 5, ICI plus VEGFR-TKI combinations and cabozantinib have improved disease control in metastatic tRCC compared with historical VEGFR-TKI monotherapy, but survival estimates remain variable across small, largely retrospective datasets [80,81,90]. Despite increasing molecular and clinical data, no MiT-RCC-specific prognostic scoring system has been externally validated, and risk stratification in practice still relies largely on conventional clinicopathologic factors.

Prognosis remains strongly influenced by stage at presentation and metastatic burden [111,112]. Within the *TFEB* alteration category, *TFEB*-amplified RCC typically arises in adults, often middle-aged to older, and behaves aggressively, with high-grade morphology and frequent metastatic presentation and disease-specific mortality [4,32,56]. By contrast, many *TFEB*-rearranged t(6;11) tumors in adolescents and young adults follow a more indolent course after complete resection, although metastatic and clinically aggressive variants have been reported [9,110,113].

At the genomic level, copy-number alterations have been linked to aggressive behavior. In molecular cohorts of tRCC, 9p21 loss (*CDKN2A*/*B*) is recurrent and has been associated with adverse outcomes, 17q gain is also recurrent but its prognostic impact appears less consistent across cohorts [15,36,47]. Additional arm-level alterations such as 22q loss, which is enriched in *ASPSCR1*::*TFE3* tumors, have also been associated with aggressive clinicopathologic features [15,36].

Proteogenomic profiling further supports biologic heterogeneity. Integrated proteogenomic analysis of tRCC identified three proteomic subtypes and three immune subtypes. The GP1/IM1-overlapping subgroup was the most aggressive and associated with poor prognosis, whereas other subtypes displayed distinct metabolic and stromal/immune programs [36]. Collectively, these data reinforce that “MiT-RCC” encompasses a spectrum of entities with markedly different natural histories.

Fusion partner identity is an important prognostic determinant within *TFE3*-rearranged RCC. Multiple cohorts have shown that *ASPSCR1*::*TFE3* tumors are enriched for high-grade morphology, nodal/distant metastases at diagnosis, and inferior outcomes compared with other *TFE3* fusions [14,15,61]. Conversely, *NONO*::*TFE3* and some other fusion partners have been associated with comparatively favorable outcomes in fusion-annotated series, recognizing that aggressive outliers occur [61]. Emerging data also suggest that *MED15*::*TFE3*-rearranged RCC represents a distinct, predominantly low-grade cystic subtype with comparatively favorable outcomes [114].

Strikingly, the fusion subtype with the worst historical prognosis may derive particular benefit from modern ICI-based combinations. In a retrospective analysis of metastatic *TFE3*-rearranged RCC treated with first-line ICI-based combination therapy, *ASPSCR1*::*TFE3* tumors had higher response rates and longer PFS than non-*ASPSCR1* fusions, although estimates are unstable given small numbers [16]. Mechanistically, *ASPSCR1*::*TFE3* tumors display a highly angiogenic, extracellular matrix-rich and proliferative transcriptomic program with recurrent 22q loss; angiogenesis and immune-activation signatures were associated with improved outcomes on ICI-based combinations, while collagen/ECM programs may attenuate the efficacy of VEGFR-TKI monotherapy [15,16]. This provides a plausible biologic explanation for an apparent paradox: the same biology that confers aggressive natural history may also create therapeutic vulnerabilities that are more effectively exploited by ICI plus VEGFR-TKI regimens.

Clinically, advanced *ASPSCR1*::*TFE3* tumors should be viewed as high-risk but potentially treatment-responsive when exposed to ICI plus VEGFR-TKI therapy, whereas other fusion types (e.g., *NONO*::*TFE3*, *SFPQ*::*TFE3*, *PRCC*::*TFE3*, *MED15*::*TFE3*) have more heterogeneous courses and, based on current evidence, appear less likely as a group to derive the same magnitude of benefit from ICI plus VEGFR-TKI combinations [16]. As molecular characterization becomes routine, future prognostic models for MiT-RCC are likely to integrate fusion partner, copy-number profile (e.g., 9p/17q/22q status), and proteogenomic subtype alongside traditional clinical factors to refine risk stratification and guide surveillance and treatment intensity.

**Table 1:** Key Clinical Studies in *TFE3*-Rearranged and *TFEB*-Altered Renal Cell Carcinoma.

Study (Year)	Design/Population	Regimen	Key Outcomes	Molecular Confirmation	*TFE3* vs. *TFEB*	Line of Therapy	Key Limitation(s)
A. Dedicated *TFE3*-/*TFEB*-rearranged RCC (tRCC) cohorts							
Boilève et al. [95]	Metastatic MiT family tRCC treated with immune checkpoint inhibitors (*n* = 24).	1L VEGFR-TKI in subset (mostly sunitinib); first ICI exposure (mostly nivolumab; some ipilimumab and ICI-based combinations).	1L TKI: ORR 10.5% (2/19), mPFS 3.0 mo. 1st ICI exposure (≥2L): ORR 16.7% (4/24), mPFS 2.5 mo.	Expert pathology review. FISH not required; FISH-negative excluded when tested.	*TFE3*: 21 (88%); *TFEB*: 3 (12%).	Mixed; first ICI exposure ≥2L (all patients).	Retrospective; small cohort; heterogeneous ICI regimens/lines; incomplete uniform molecular confirmation; no fusion-partner annotation.
Thouvenin et al. [81]	Metastatic MiT family tRCC treated with cabozantinib (*n* = 52 evaluable).	Cabozantinib (various lines).	ORR 17.3% (9/52; 2 CR, 7 PR); mPFS 6.8 mo; mOS 18.3 mo; durable clinical benefit 46.2%.	Suggestive morphology + nuclear *TFE3*/*TFEB* IHC in all. FISH available/positive in 40/52 (77%). Fusion partner reported for subset (*n* = 10).	*TFE3*: 46 (88.5%); *TFEB*: 6 (11.5%).	Cabozantinib 1L: 11 (21%); 2L: 15 (29%); ≥3L: 26 (50%).	Retrospective; heterogeneous prior therapy; not uniformly FISH-confirmed; limited fusion-partner data.
Alhalabi et al. [80]	Adult advanced tRCC (*TFE3*- or *TFEB*-rearranged) (*n* = 29).	ICI + VEGF/VEGFR-targeted therapy (*n* = 11) vs. dual ICI (*n* = 18; mostly nivolumab/ipilimumab).	ICI + TKI: ORR 36%, mPFS 5.4 mo, mOS 30.7 mo. Dual ICI: ORR 5.6% (1/18), mPFS 2.8 mo, mOS 17.8 mo.	FISH-confirmed *TFE3* or *TFEB* rearrangement in all included cases.	*TFE3*: 22; *TFEB*: 7.	Mixed; 59% received an ICI-based combination in 1L.	Retrospective; small sample; heterogeneous regimens and lines; no fusion-partner annotation.
Zhao et al. [16]	Metastatic *TFE3*-rearranged RCC (*n* = 38); fusion-partner analysis in subset.	ICI-based combination therapy; outcomes stratified by fusion partner.	In 1L ICI-combination cohort with known fusion partner (*n* = 18): *ASPSCR1*::*TFE3* ORR 62.5% (5/8), mPFS not reached vs. non-*ASPSCR1* ORR 10.0% (1/10), mPFS 6.5 mo.	*TFE3* fusion partner defined by sequencing (RNA-based fusion detection).	*TFE3* only (100%).	Primarily 1L ICI-combination subgroup analysis (overall cohort lines mixed).	Retrospective; small fusion-annotated subset; potential selection bias; estimates unstable. (Note: abstract reports ORR 67.5% vs. 10.0%, but main text provides 5/8 = 62.5%.)
Ged et al. [90]	Metastatic *TFE3*-rearranged RCC treated with ICI combinations (*n* = 22).	ICI + VEGF-TKI (*n* = 14) vs. dual ICI (*n* = 8).	ICI + TKI: ORR 54% (6/11 evaluable), median TTF 6.2 mo. Dual ICI: ORR 14% (1/7 evaluable), median TTF 1.2 mo. Median OS numerically longer in dual ICI (36.7 vs. 15.6 mo; NS).	FISH-confirmed *TFE3* rearrangement (per report).	*TFE3* only.	Mixed lines (first ICI-combination exposure).	Retrospective; small cohort; heterogeneous agents; OS confounded by subsequent therapy; response denominators based on evaluable patients.
AREN1721 [94]	Unresectable/metastatic *TFE3*- or *TFEB*-rearranged RCC across ages (15 enrolled; 13 eligible; median age 16).	Randomized: nivolumab + axitinib vs. axitinib vs. nivolumab.	Combination vs. nivolumab: ORR 33% (2/6) vs. 0% (0/5); mPFS 10.5 vs. 1.8 mo; OS favored combo (*p* = 0.003).	Molecularly defined eligibility (rearranged *TFE3*/*TFEB*); confirmation method not detailed in abstract.	Not reported.	Treatment-naïve for metastatic setting (per trial design); very small axitinib-alone arm.	Abstract only; underpowered; pediatric/AYA-skewed; very small arms → unstable estimates.
B. Prospective non-clear-cell RCC trials including translocation RCC subsets							
Lee et al. [87]	Phase II non-clear-cell RCC (cohort 1; *n* = 40) including translocation-associated RCC (*n* = 2).	Cabozantinib + nivolumab.	Overall cohort 1: ORR 47.5%, mPFS 12.5 mo, mOS 28 mo. tRCC subset: 1/2 confirmed PR (ORR 50%).	Investigator-assessed histology; molecular confirmation not reported for tRCC subset.	Not reported.	0–1 prior lines allowed; no prior ICI (per trial).	tRCC subset extremely small; not powered for subtype conclusions.
Albiges et al. KEYNOTE-B61 [85,86]	Phase II 1L non-clear-cell RCC (*n* = 158) including investigator-assessed tRCC (*n* = 6).	Pembrolizumab + lenvatinib (1L).	Overall: confirmed ORR 49–51% (updated analysis); mPFS 17.9 mo; OS not reached. tRCC subset: ORR 66.7% (4/6); DCR 83.3% (5/6).	Investigator-assessed tRCC; fusion partner and molecular confirmation not reported for subset.	Not reported.	Strict 1L.	Signal-seeking only; very small tRCC subset; no fusion-partner annotation.
Bergmann et al. [96]	Randomized phase II 1L non-clear-cell RCC; translocation RCC subgroup present (~16–17; outcomes not reported separately).	Nivolumab + ipilimumab vs. standard of care (predominantly VEGFR-TKI; some ICI + TKI depending on SOC).	Overall trial: 12-mo OS 78% vs. 68% (primary endpoint); ORR 32.8% vs. 19.3%; PFS HR 0.99; treatment discontinuation 17% vs. 9%. tRCC outcomes not reported separately.	Central pathology review; molecular confirmation not specified for subgroup.	Not reported.	Strict 1L.	Subtype analyses limited; SOC arm heterogeneous; tRCC outcomes not reported separately.

Note: These clinical studies predominantly include fusion-driven tRCC (*TFE3*- or *TFEB*-rearranged). *TFEB*-amplified RCC is largely underrepresented in prospective and retrospective systemic-therapy series. Study design and key limitations are shown to contextualize evidence strength; outside the small randomized or prospective subsets, comparative efficacy signals should be interpreted as hypothesis-generating. Abbreviations: 1L, first-line; 2L, second-line; AYA, adolescent and young adult; CR, complete response; DCR, disease control rate; FISH, fluorescence in situ hybridization; ICI, immune checkpoint inhibitor; mOS, median overall survival; mPFS, median progression-free survival; NS, not statistically significant; ORR, objective response rate; OS, overall survival; PFS, progression-free survival; PR, partial response; RCC, renal cell carcinoma; SOC, standard of care; TKI, tyrosine kinase inhibitor; tRCC, translocation renal cell carcinoma; TTF, time to treatment failure; VEGFR, vascular endothelial growth factor receptor.

## 7. Conclusions and Future Directions

MiT-RCC is best viewed as a spectrum of molecularly defined renal cancers rather than a single entity. Subclassifying tumors as *TFE3*-rearranged, *TFEB*-rearranged, or *TFEB*-amplified, and annotating the *TFE3* fusion partner when feasible, helps explain the wide range of clinical behavior observed in practice and is increasingly relevant for prognosis, clinical-trial eligibility, and emerging treatment selection. Notably, *ASPSCR1*::*TFE3* tumors carry an adverse baseline prognosis, yet fusion-annotated retrospective cohorts suggest they may derive disproportionate benefit from ICI plus VEGFR-TKI combinations. This hypothesis-generating observation is consistent with the angiogenic/stromal and immune-related programs reported in this fusion subtype and warrants confirmation in larger, prospectively captured datasets.

Diagnostic practice is also evolving beyond reliance on *TFE3*/*TFEB* immunohistochemistry alone. Integrating morphology with supportive IHC surrogates, break-apart FISH, sequencing-based fusion detection, and copy-number assessment for *TFEB* amplification improves sensitivity for cryptic events and enables clinically actionable subclassification. This is particularly important within *TFEB*-altered tumors, where distinguishing rearrangement from amplification carries distinct prognostic and therapeutic implications.

From a therapeutic standpoint, the available evidence, although limited by small and heterogeneous cohorts with predominantly retrospective designs, most consistently supports ICI plus VEGF/VEGFR-targeted combinations as the first-line systemic approach for eligible patients with metastatic fusion-driven translocation RCC. Cabozantinib has a comparatively strong evidence base in later lines and remains a key option when not incorporated into upfront combination strategies. In contrast, cohort-level activity of ICI monotherapy or dual ICI appears modest, and optimal systemic management of *TFEB*-amplified RCC remains uncertain, underscoring the importance of clinical-trial enrollment whenever feasible. In pediatric and adolescent patients, the early closure of the only dedicated randomized trial (AREN1721) due to poor accrual highlights the difficulty of generating prospective evidence in rare childhood cancers and supports international cooperative networks and age-inclusive trial designs that balance efficacy assessment against long-term toxicity.

Several priorities follow. First, prospective trials and multi-institutional registries that include MiT-RCC rather than excluding it are essential, with standardized molecular confirmation and harmonized reporting of fusion partners and *TFEB* copy-number status. Second, biomarker-driven strategies should be developed to account for molecular heterogeneity, including angiogenic/stromal programs, *NRF2*-associated stress-response states, condensate biology, and proteogenomic/immune subtypes. Third, emerging vulnerabilities such as oxidative metabolism dependencies (e.g., *PPARGC1A*/PGC-1α-driven programs) and related preclinical nodes including *EGLN1*/PHD2-HIF signaling, as well as rational pathway co-targeting strategies (e.g., VEGF/VEGFR with mTOR signaling), warrant clinical translation in prospective, molecularly annotated, fusion-aware combination trials. Overall, MiT-RCC illustrates how precision oncology in rare RCC begins with molecularly precise diagnosis and will advance through collaborative, molecularly annotated clinical research.

## Data Availability

No new data were created or analyzed in this study. Data sharing is not applicable to this article.
